# MiR-135a-5p suppresses trophoblast proliferative, migratory, invasive, and angiogenic activity in the context of unexplained spontaneous abortion

**DOI:** 10.1186/s12958-022-00952-z

**Published:** 2022-05-24

**Authors:** Yebin Lu, Xiaoli Zhang, Xueyu Li, Lingjie Deng, Changqiang Wei, Dongmei Yang, Xuemei Tan, Weicheng Pan, Lihong Pang

**Affiliations:** 1 Department of Prenatal Diagnosis and Genetic Diseases, First Affiliated Hospital of Guangxi Medical University, Guangxi, China; 2 Guangxi Medical University, Guangxi, China; 3 Department of Obstetrics and Gynecology, Fourth Affiliated Hospital of Guangxi Medical University, Liuzhou, China; 4 Maternal and Child Health Hospital of Guangxi Zhuang Autonomous Region, Guangxi, China

**Keywords:** Unexplained spontaneous abortion (SA), miR-135a-5p, PTPN1(PTP1B), Trophoblast

## Abstract

**Background:**

Spontaneous abortions (SA) is amongst the most common complications associated with pregnancy in humans, and the underlying causes cannot be identified in roughly half of SA cases. We found miR-135a-5p to be significantly upregulated in SA-associated villus tissues, yet the function it plays in this context has yet to be clarified. This study explored the function of miR-135a-5p and its potential as a biomarker for unexplained SA.

**Method:**

RT-qPCR was employed for appraising miR-135a-5p expression within villus tissues with its clinical diagnostic values being assessed using ROC curves. The effects of miR-135a-5p in HTR-8/SVneo cells were analyzed via wound healing, Transwell, flow cytometry, EdU, CCK-8, and tube formation assays. Moreover, protein expression was examined via Western blotting, and interactions between miR-135a-5p and PTPN1 were explored through RIP-PCR, bioinformatics analyses and luciferase reporter assays.

**Results:**

Relative to normal pregnancy (NP), villus tissue samples from pregnancies that ended in unexplained sporadic miscarriage (USM) or unexplained recurrent SA (URSA) exhibited miR-135a-5p upregulation. When this miRNA was overexpressed in HTR-8/SVneo cells, their migration, proliferation, and cell cycle progression were suppressed, as were their tube forming and invasive activities. miR-135a-5p over-expression also downregulated the protein level of cyclins, PTPN1, MMP2 and MMP9. In RIP-PCR assays, the Ago2 protein exhibited significant miR-135a-5p and PTPN1 mRNA enrichment, and dual-luciferase reporter assays indicated PTPN1 to be a bona fide miR-135a-5p target gene within HTR-8/SVneo cells.

**Conclusion:**

miR-135a-5p may suppress trophoblast migratory, invasive, proliferative, and angiogenic activity via targeting PTPN1, and it may thus offer value as a biomarker for unexplained SA.

**Supplementary Information:**

The online version contains supplementary material available at 10.1186/s12958-022-00952-z.

## Background

Miscarriage, also referred to as spontaneous abortion (SA), is among the most common complications associated with human pregnancy, and is generally defined as an intrauterine pregnancy loss before viability [1]. In patients with SA, recurrent spontaneous SA (RSA) is diagnosed when patients experience two or more SAs, otherwise the cases are diagnosed as sporadic miscarriage (SM) [2–4]. An estimated 15–25% of clinical pregnancies ultimately end in SA [1], with 2–5% of these corresponding to cases of RSA [5], which can have a strong negative impact on the physical and mental health of affected women. The causes of SA generally cannot be identified in roughly half of cases [2], and no reliable prognostic or diagnostic biomarkers associated with this pathological pregnancy are available at present. So far, SA is diagnosed through high-resolution dynamic transvaginal ultrasonography, and pregnancy termination is the only treatment available following SA diagnosis [2, 3, 5]. Thus, there is an urgent need to more fully clarify the underlying mechanisms of unexplained SA and find better diagnostic biomarkers associated with this pregnancy complication.

MicroRNAs (miRNAs) are small RNAs lacking coding potential that serve as key post-transcriptional regulators in a range of physiological and pathological settings [6, 7], and they can regulate the expression of roughly 30% of genes in various contexts [8–10]. Importantly, miRNAs exhibit relatively good stability in biofluids and tissue samples, highlighting the potential utility as prognostic or diagnostic biomarkers in many diseases such as cancer [11, 12], inflammation [13], and cardiovascular diseases [14], with certain miRNAs having further been identified as targets for preclinical therapeutic intervention [14]. A considerable number of investigations suggest that abundant miRNA expression is detectable within the placental villus, decidua, and peripheral blood of humans, wherein these non-coding RNAs (ncRNAs) may contribute to SA pathogenesis by disrupting the function of extravillous trophoblasts [15–17], highlighting their potential value as biomarkers of SA [18–20].

MiR-135a-5p is a highly conserved miRNA that has previously been shown to be upregulated in cardio-cerebral ischemic diseases, functioning to suppress ischemia-reperfusion-related injuries [21, 22]. In cancer, it has variably been suggested to act as an oncogene [23–25] or tumor suppressor [26, 27], modulating tumor migration, proliferation, and invasion [28]. The mechanism through which miR-135a-5p may contribute to SA incidence remain to be fully defined. In our prior transcriptomic sequencing investigation, we discovered miR-135a-5p to be substantially upregulated in villus tissue samples from unexplained SA cases as compared to those from normal pregnancies (NP). Consistently, another report has demonstrated miR-135a upregulation in samples of plasma from women that experienced early loss of pregnancy [29]. Petracco et al. further detected endometrial miR-135a expression in women suffering from endometriosis and posited that such upregulation is associated with the suppression of implantation-related genes [30]. Moreover, Zhao et al. determined that miR-135 upregulation was sufficient to inhibit the activation of the NLRP3 inflammasome, thereby attenuating preeclampsia-related inflammatory responses [31]. This is noteworthy that inflammation is critical to embryo implantation and an intense maternal inflammatory reaction is observed upon implantation [32–34]. Extravillous trophoblasts are also similar to cancer cells in many aspects, exhibiting a high degree of proliferative, invasive, and migratory activity [35]. We thus hypothesized that miR-135a-5p may represent a biomarker associated with SA and may also directly alter trophoblast function, thereby contributing to the pathogenesis of SA.

PTPN1 (Protein Tyrosine Phosphatase Non-Receptor Type 1) is an important regulator of biological functions of invasion, migration, proliferation, and apoptosis [32, 33]. In myocardial ischemia/reperfusion, miR-135a-5p was revealed to downregulate PTPN1 and have protective effects [22]. However, contributions by miR-135a-5p/PTPN1 axis within miscarriage, has yet to be determined.

In light of the above hypothesis, this study sought to detect the expression of miR-135a-5p in the villus specimens from unexplained SA cases and used a series of in vitro assays to analyze its impact on trophoblasts. Together, our results revealed that upregulation of miR-135a-5p impaired trophoblast functionality, potentially contributing to SA pathogenesis and highlighting its promise as a biomarker for this pregnancy complication.

## Methods

### Identification of miRNAs associated with SA

SA-related miRNAs were identified in our RNA-sequencing data set, the details of which are shown in Table S1 in the additional files. Differential expression screening criteria were: |log2FC| > 2.0 and *p* < 0.05. Some genes were visualized via a heat map.

### Villus sample collection

Placental villous tissue from women in the first-trimester of pregnancy were harvested between December 2019 and June 2020 at First Affiliated Hospital of Guangxi Medical University. Women eligible for this study were those with ultrasound-confirmed pregnancies and obstetric examinations having < 12-week gestational age. The exclusion criteria included known causes of miscarriage such as uterine abnormalities, infectious diseases, adverse life history, occupational hazards, or chromosomal abnormalities. The diagnostic criteria were as follows: when no fetal heartbeat was detected following multiple ultrasound examinations and the uterine size was smaller than expected for gestational age, this was diagnosed as a SA. Two or more instances of SA are diagnosed as RSA, while SM is otherwise diagnosed. This study ultimately incorporated first-trimester placental villous tissue samples from normal pregnancies (NP, *n* = 50) and unexplained SA cases (n = 50), with the latter group consisting of URSA (*n* = 18) and USM (*n* = 32) patients. Gestational age and maternal age in the unexplained SA and NP groups were strictly matched. Corresponding clinical data are compiled in Table S2.

### Cell culture

HTR-8/SVneo (Zhongqiao Xinzhou, Shanghai, China) were cultivated within RPMI-1640 (Gibco, USA), supplied with 10% FBS (Sijiqing, Zhejiang, China) and 1% penicillin/streptomycin at 37 °C in a 5% CO_2_ incubator.

### Transfection

miR-135a-5p mimics and the related negative control (NC) were procured through Gene Pharma (Shanghai, China), with sequences being compiled in Supplementary Table S3. Lipofectamine 3000 (Invitrogen, USA) was employed for the transfection of these constructs into cells when 60–70% confluent based on provided directions.

### RT- qPCR

The extraction of total RNA was executed with Trizol (TaKaRa, Japan), following which a PrimeScript RT Reagent Kit (TaKaRa, Japan) was employed for generating cDNA. The expressions of miR-135a-5p and other genes were assessed with the SYBR Premix ExTaq, with U6 or β-actin serving as reference controls and relative expression being determined through 2^-△△Ct^ methodology. Primers are compiled in Table S4.

### Western blotting

Total protein was extracted from cells using RIPA buffer (Beyotime, China) containing PMSF (Beyotime, China). Samples were consequently segregated through SDS-PAGE, and transported onto polyvinylidene difluoride (PVDF) membranes, followed by 5% non-fat milk blocking (120 minutes/RT), with eventual primary and secondary antibody staining before imaging.

Primary antibodies were specific for PTPN1 (1:5000; Proteintech, China), MMP2 (1:500; Cell Signaling Technology, USA), MMP9 (1:2000; Boster, China), cyclin D1 (1:15000; Proteintech, Wuhan, China), cyclin B1 (1:2000; SAB, United States), E-cadherin (1:1000; Proteintech, Wuhan, China), and Vimentin (1:40,000; Proteintech, Wuhan, China). Antibodies for GAPDH (1:50,000; Proteintech, Wuhan, China) or β-Tubulin (1:50,000; Proteintech, Wuhan, China) served as internal controls. Secondary horseradish peroxidase (HRP)-conjugated goat anti-rabbit IgG (H + L) (1:20000; Abcam, UK) together with Goat anti-murine IgG (H + L) antibody (1:40000; Invitrogen, USA) were used. ImageJ was used for densitometric analyses, with GAPDH or β-Tubulin being used for normalization.

### CCK-8 assay

Transfected cells were added to the plates containing 96 wells (1.5 × 10^3^/well) and cultured under standard conditions for 4 days, with 100 μL/well of CCK-8 solution (Dojindo, Kumamoto, Japan) (1:10) then being added to the media in each well. Following an additional 3 h incubation, absorbance within individual wells were assessed (450 nm) via microplate-reader.

### Flow cytometry

Following 48 h from transfection, cells were fixed (using 75% ethanol at − 20 °C), resuspended in phosphate-buffered saline (PBS) and stain treated through Cell Cycle Detection Kit (MultiSciences, China), followed by flow cytometry analyses, with the results being assessed using FlowJo V10.

### 5-Ethynyl-2′-deoxyuridine (EdU) assay

HTR-8/SVneo cell cultures were accumulated upon 48 hours after transfection, added to 96-well-plates (5.0 × 10^3^/well) followed by culture for an additional 36 h. They were incubated with EdU reagent (1:1000), fixed with 4% paraformaldehyde, and stained with the EdU kit (Ribobio, Guangzhou, China) based on provided directions, followed by imaging with a fluorescence inverted microscope (Olympus, Japan) at 200x. Cells were counted using ImageJ to determine the cellular proliferation rate.

### Wound healing assay

Migration was assessed using the wound healing assay. Cells were added to the plates containing 6 wells (2 × 10^5^/well) and a 100 μL pipette tip was used to scratch the cell monolayer at 48 h after transfection. Fresh serum-free media was then added and cells were imaged at 0, 24 and 48 h to assess wound healing area with an inverted microscope (Olympus, Japan) at 100x.

### Transwell assay

HTR-8/SVneo cells migratory and invasive activities were scrutinized via Transwell assay. In migration assessments, 5 × 10^4^ cells per well were resuspended in serum-free media in an upper Transwell insert chamber, whereas lower chambers were occupied with media including 20% FBS. Following 36 h, cells were fixated using 4% paraformaldehyde, followed by 0.1% crystal violet-staining. Stained cells were observed through inverted microscopy (Olympus, Japan) at 200x. In invasion assessments, 1 × 10^5^ cells were seeded in upper Matrigel-coated upper chambers (8um pores; Corning, NY, USA) followed by a 48-h incubation, with all other conditions being the same as for migration assays.

### Tube formation assay

The HTR-8/SVneo extravillous trophoblast cell line was employed for tube formation assays for the analysis of angiogenesis. Matrigel was thawed at 4 °C and added into pre-chilled 96-wells plate (75 μL/well), after which it was incubated at 37 °C for 60 min to form a solid. Cells were resuspended in serum-free media at 1.8 × 10^5^ cells/well. Following incubation for 6 h, tube formation was assessed and imaged with an inverted microscope (Olympus, Japan) at 100x.

### Bioinformatics analyses of SA-related miRNAs and genes

The miRWalk (http://mirwalk.umm.uni-eidelberg.de/), ENCORI (https://starbase.sysu.edu.cn/index.php), TargetScan (http://www.targetscan.org/vert_72/), and present sequencing data set were used for the potential identification of target genes, with a Venn online assessment (https://bioinfogp.cnb.csic.es/tools/venny/index.html) being used for target overlapping.

### Argonaute 2-RNA immunoprecipitation

RIP measurement was conducted through Magna RNA Immunoprecipitation Kit (Millipore, USA) based on provided instructions. HTR-8/SVneo cells were collected and lysed, then incubated (4 °C /overnight) through Magnetic beads conjugated with anti-Argonaute 2 (AGO2) or control anti-immunoglobulin G (IgG). Finally, RT-qPCR was conducted to assess PTPN1 and miR-135a-5p relative fold enrichment. The primer sequences used for this assay are provided in Supplementary Table S4.

### Dual-luciferase reporter assay

Bioinformatics analyses was used to predict the binding across PTPN1 and miR-135a-5p. Wild-type (WT) / mutated (MUT) versions for 3′-untranslated region (3′-UTR) of PTPN1 harboring miR-135a-5p bonding regions were inserted within luciferase reporter-vector constructs, with the MUT version being employed for confirming the specificity for PTPN1 and miR-135a-5p interactions. HTR-8/SVneo cultures were co-transfected using WT-PTPN1 or MUT-PTPN1 together with miR-135a-5p-mimics or NC-mimics. Following co-transfection for 48 h, the binding activity were determined through dual-luciferase reporter assessment kit (Promega, USA), with results being calculated based on relative luciferase activity levels.

### Statistical analysis

SPSS v.20.0 was implemented upon all statistics-based assessments, while data were analyzed with GraphPad Prism 7 and images were analyzed with ImageJ, with AI being used for image combinations. Outcomes are given as means ± SEM from at least three independent analyses, and were compared via two-tailed t-tests. Receiver operating characteristic (ROC) curve analyses were utilized to gauge diagnostic utility, and *P* < 0.05 was the threshold of significance.

## Results

**Fig. 1:**
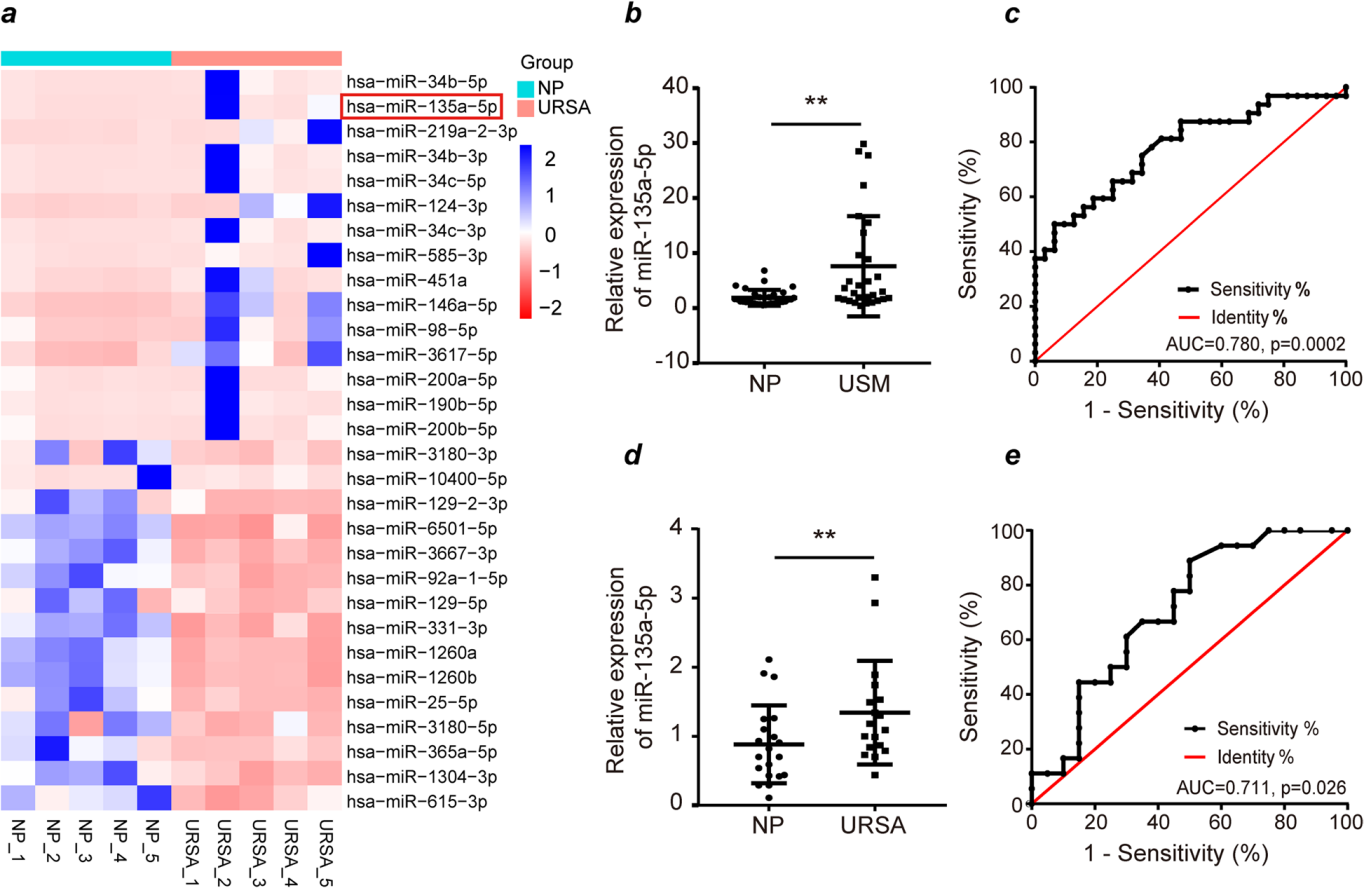
miR-135a-5p offers promise as a diagnostic biomarker for unexplained SA. (**a**) Heat maps demonstrating relative miR-135a-5p expression in first-trimester villous tissue samples from NP (*n* = 5) and URSA (n = 5) patients. (**b**) MiR-135a-5p expression in the villous tissues from NP (*n* = 32) and USM (n = 32) patients. (**c**) MiR-135a-5p offered good diagnostic potential for USM. (**d**) MiR-135a-5p expression in the villous tissues from NP (*n* = 18) and URSA (n = 18) patients. (**e**) MiR-135a-5p offered good diagnostic potential for URSA. NP: normal pregnancy; SA: spontaneous abortion; USM: unexplained sporadic miscarriage; URSA: unexplained recurrent spontaneous abortion; ROC: Receiver operating characteristic; AUC: Area under the curve. *P < 0.05, **P < 0.01, *** *P* < 0.001

Our prior transcriptomic sequencing data revealed miR-135a-5p expression to be significantly increased (Fig. 1a, Table S1), suggesting a potential role for this miRNA as a regulator of SA incidence.

### MiR-135a-5p exhibits promise as a diagnostic biomarker for unexplained SA

No differences in gestational age, maternal age, body mass index (BMI) or history of gestation were observed when comparing the unexplained SA (*n* = 50) and NP (*n* = 50) groups (*P* > 0.05; Table S2). Subsequent RT-qPCR analyses of tissue samples from these two groups revealed marked upregulation for miR-135a-5p in villous tissue from both USM and URSA patients relative to NP patients (*P* < 0.05, Fig. 1b, d). ROC curves were further implemented to gauge the potential diagnostic utility of miR-135a-5p as a predictor of USM and URSA, yielding respective area-under-curve (AUC) values of 0.780 (95% CI: 0.613–0.810, *P* = 0.0002, Fig. 1c) and 0.711 (95%CI 0.546–0.876, *P* = 0.026, Fig. 1e) for USM and URSA, respectively. As such, while it may be more important in USM than URSA, miR-135a-5p may act as a predictor of unexplained SA and offers good diagnostic potential.

### MiR-135a-5p mimics suppress proliferation and cell cycle progression in HTR-8/SVneo cells

**Fig. 2:**
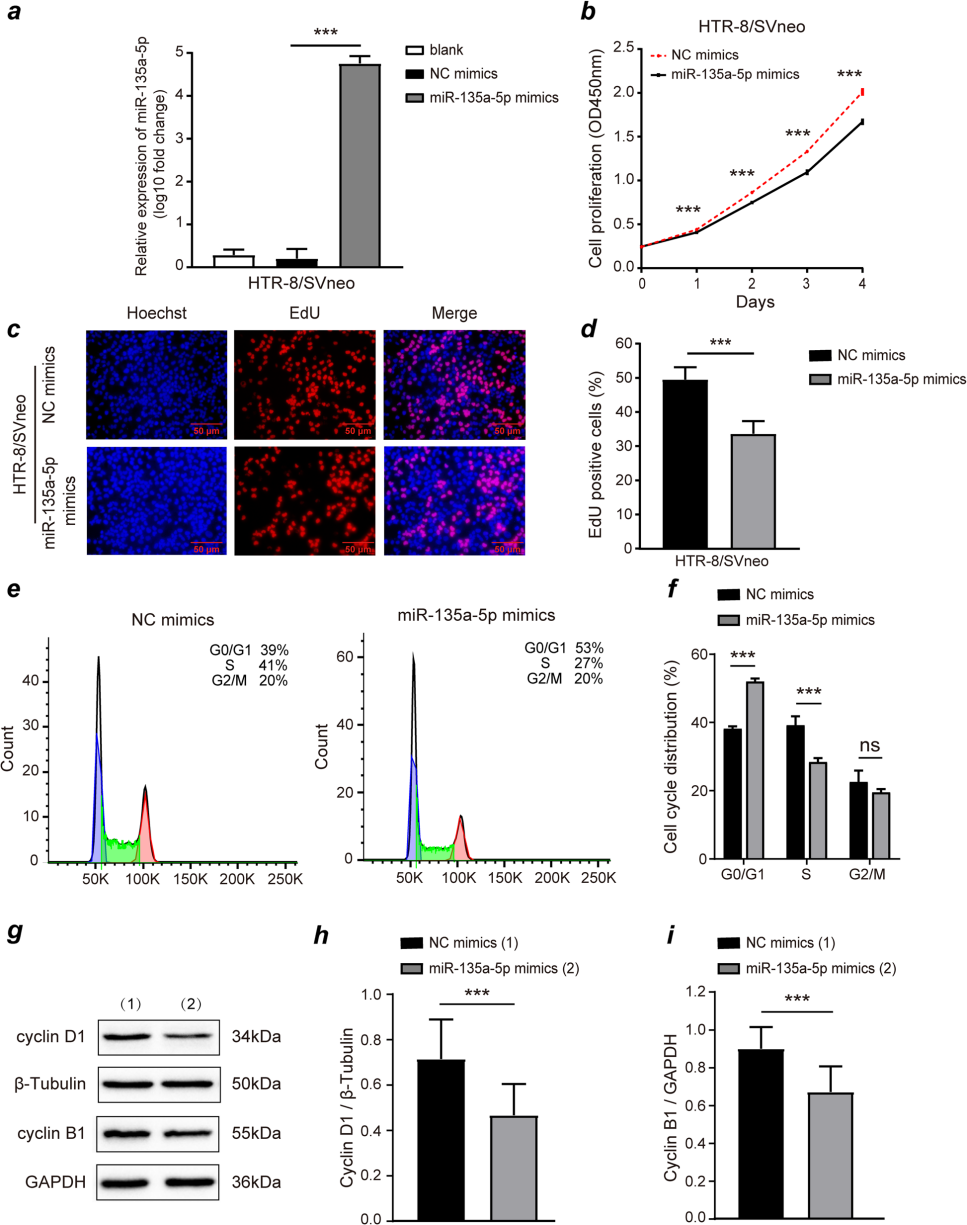
MiR-135a-5p mimics inhibit HTR-8/SVneo cell proliferation and cell cycle progression. (a) MiR-135a-5p expression in HTR-8/SVneo following miR-135a-5p mimics or NC mimics transfection. (b) MiR-135a-5p overexpression inhibited cell proliferation in CCK-8 assays, with similar results in EdU assays (c-d). (e-f) MiR-135a-5p overexpression inhibited G0/G1 to S phase progression of the cell cycle within HTR-8/SVneo cells. (g-i) Cyclin D1 and cyclin B1 protein levels were reduced following the overexpression of miR-135a-5p within HTR-8/SVneo cells, with GAPDH and β-Tubulin serving as reference controls. Outcomes are means ± SD from at least three experiments. NC: negative control; CCK-8: Cell counting Kit-8; EdU: 5-Ethynyl-2′-deoxyuridine. *P < 0.05, **P < 0.01, *** *P* < 0.001

HTR-8/SVneo cells were transfected with miR-135a-5p mimics to upregulate the expression of miR-135a-5p, and RT-qPCR analyses were employed to confirm transfection efficiency. The results revealed that miR-135a-5p mimics significantly increased miR-135a-5p levels as compared to NC transfection in HTR-8/SVneo cells (*P* < 0.05, Fig. 2a). Then, A CCK-8 assay was used for monitoring cell viability, revealing miR-135a-5p mimics markedly impair the viability of HTR-8/SVneo cells relative for NC mimics transfection (*P* < 0.05, Fig. 2b). EdU assays further highlighted miR-135a-5p mimics transfection markedly decreased frequency for EdU-positive cells (*P* < 0.05, Fig. 2c-d), consistent with the inhibition of DNA replication. When flow-cytometry was used for examining cell-cycle distributions in HTR-8/SVneo cells, miR-135a-5p mimics transfection revealed an increased degree of cells within G0/G1 phase, whereas frequency for S phase cells was reduced, and no significant alterations in G2/M-phase cells was denoted (*P* < 0.05, Fig. 2e-f). Consistent with the suppression of G0/G1 to S phase progression, after miR-135a-5p mimics transfection, the expression of CyclinD1, which is integral to the G0/G1 to S phase transition, was reduced by roughly 35%. And with a corresponding drop in mitosis-related CyclinB1 expression (*P* < 0.05, Fig. 2g-i), whose changes were in lines with a slight drop in G2/M phase frequency, yet no significant changes in cell cycle distribution was detected by flow cytometry. Overall, these results suggesting miR-135a-5p could thwart HTR-8/SVneo cellular proliferation and the cell cycle progression.

### MiR-135a-5p mimics suppress the migration and invasion of HTR-8/SVneo cells

**Fig. 3:**
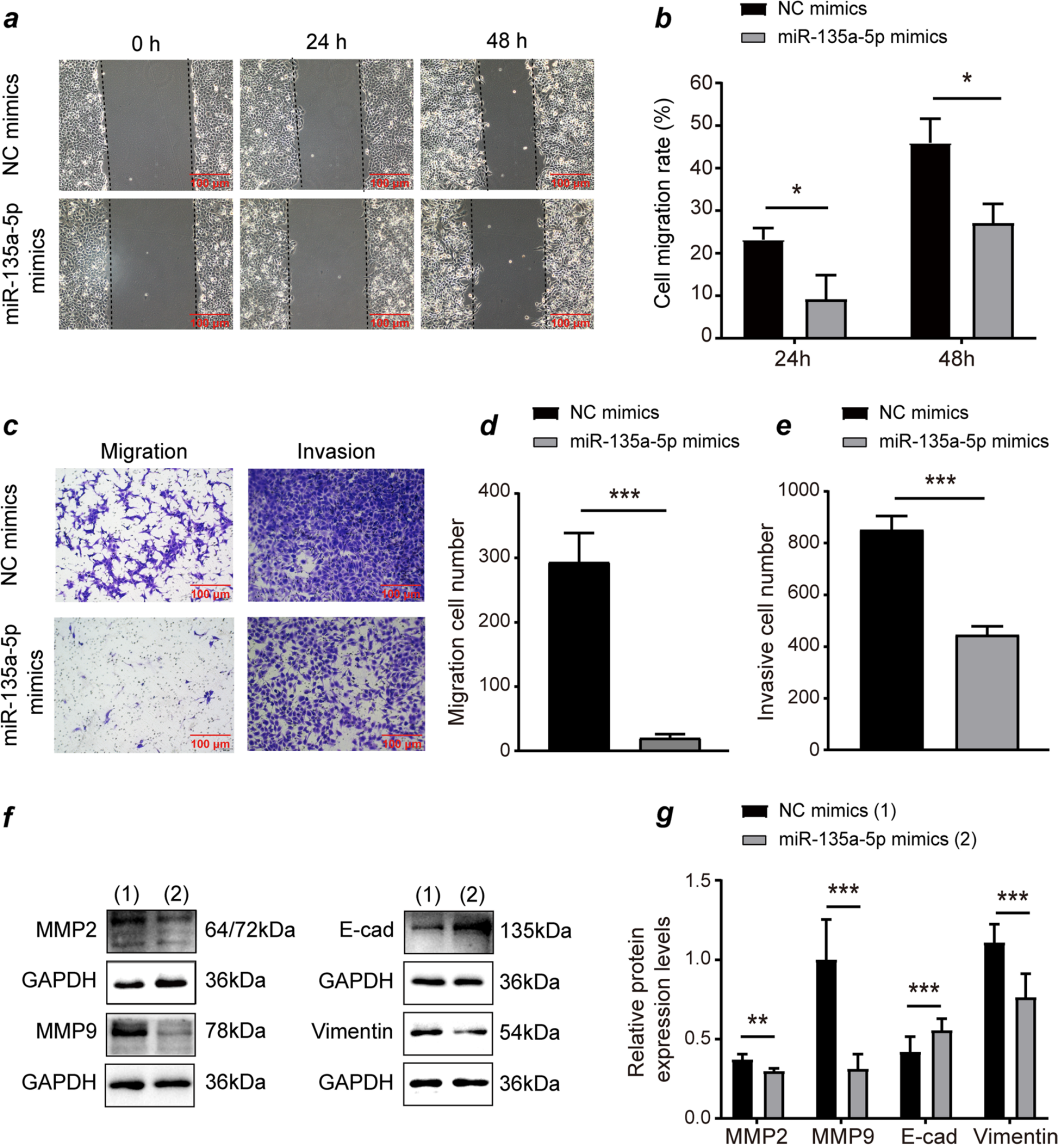
miR-135a-5p mimics suppress migratory and invasive activity in HTR-8/SVneo cells. (**a**-**b**) Overexpressing miR-135a-5p inhibited HTR-8/SVneo cell migration in a wound healing assay. (**c**-**e**) Overexpressing miR-135a-5p suppressed HTR-8/SVneo cell migration and invasion in Transwell assessments. (**f**-**g**) Western blotting revealed E-cad upregulation and the downregulation of MMP2, MMP9, and Vimentin, with GAPDH as a reference control. Data are means ± SD from at least three experiments. E-cad: E-cadherin. *P < 0.05, **P < 0.01, *** *P* < 0.001

Trophoblast migration and invasion is tied to embryo implantation and placental development. As such, any alterations in trophoblast invasion and migratory activity may contribute to the pathogenesis of unexplained SA. Wound healing assays were therefore utilized to explore HTR-8/SVneo migration and invasive properties, revealing that miR-135a-5p mimics-transfection reduced HTR-8/SVneo cell migration (*P* < 0.05, Fig. 2a-b). Transwell migration assays further confirmed these results in HTR-8/SVneo cells (P < 0.05, Fig. 3c-d), with Transwell invasion assays revealing miR-135a-5p mimics to significantly attenuate cellular invasion by roughly 47.64% (*P* < 0.05, Fig. 3c, e). Moreover, miR-135a-5p mimics markedly reduced MMP2, MMP9, and vimentin protein expression, while upregulating E cadherin (Fig. 3). These data support a model wherein miR-135a-5p is able to negatively modulate HTR-8/SVneo cell invasion and migration.

### MiR-135a-5p mimics suppress angiogenesis in HTR-8/SVneo cells

**Fig. 4:**
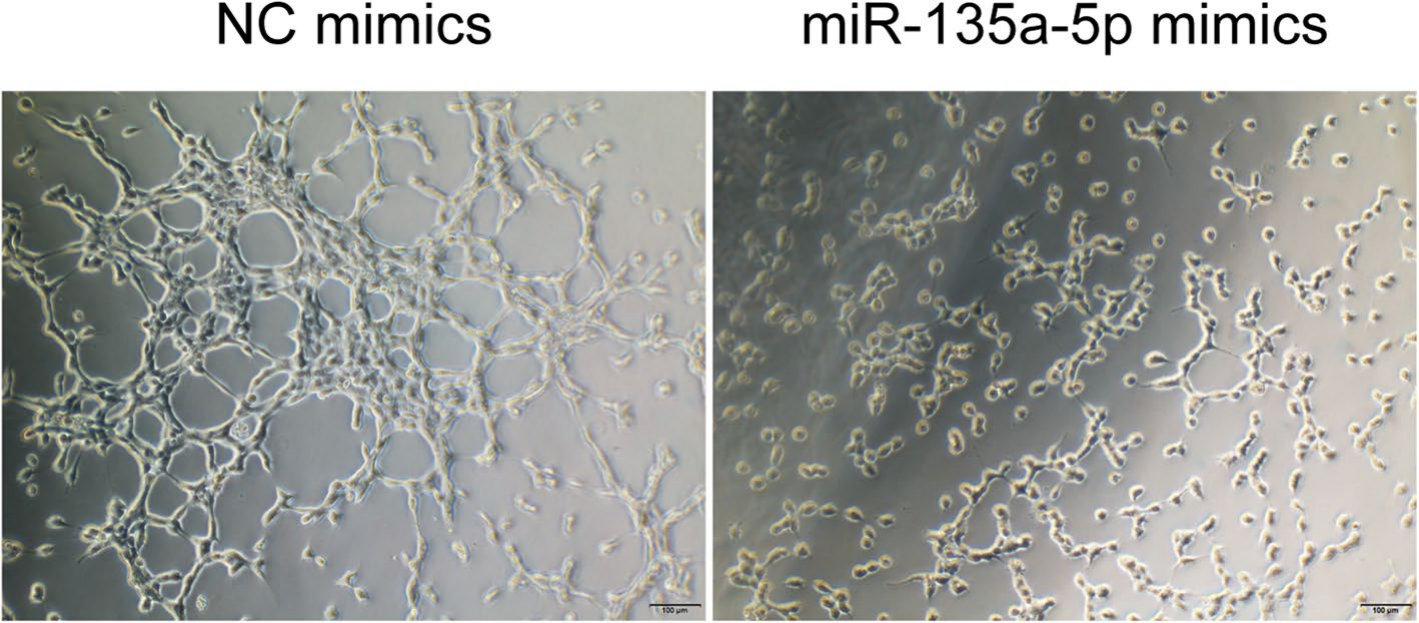
miR-135a-5p overexpression suppresses HTR-8/SVneo cell angiogenic activity. The overexpression of miR-135a-5p was found to inhibit HTR-8/SVneo cell angiogenic activity in a tube forming assay

Given that insufficient angiogenesis is closely tied to the pathogenesis of SA and extravillous trophoblast perform a role in uterine spiral artery transformation, we appraised the impact of miR-135a-5p on angiogenic in HTR-8/SVneo cells via a tube formation assay. This assay divulged miR-135a-5p mimics transfection reduced vascular network formation (Fig. 4). As such, miR-135a-5p may contribute for SA incidence through inhibition of angiogenesis.

### PTPN1 acts as target for miR-135a-5p within HTR-8/SVneo cells

**Fig. 5:**
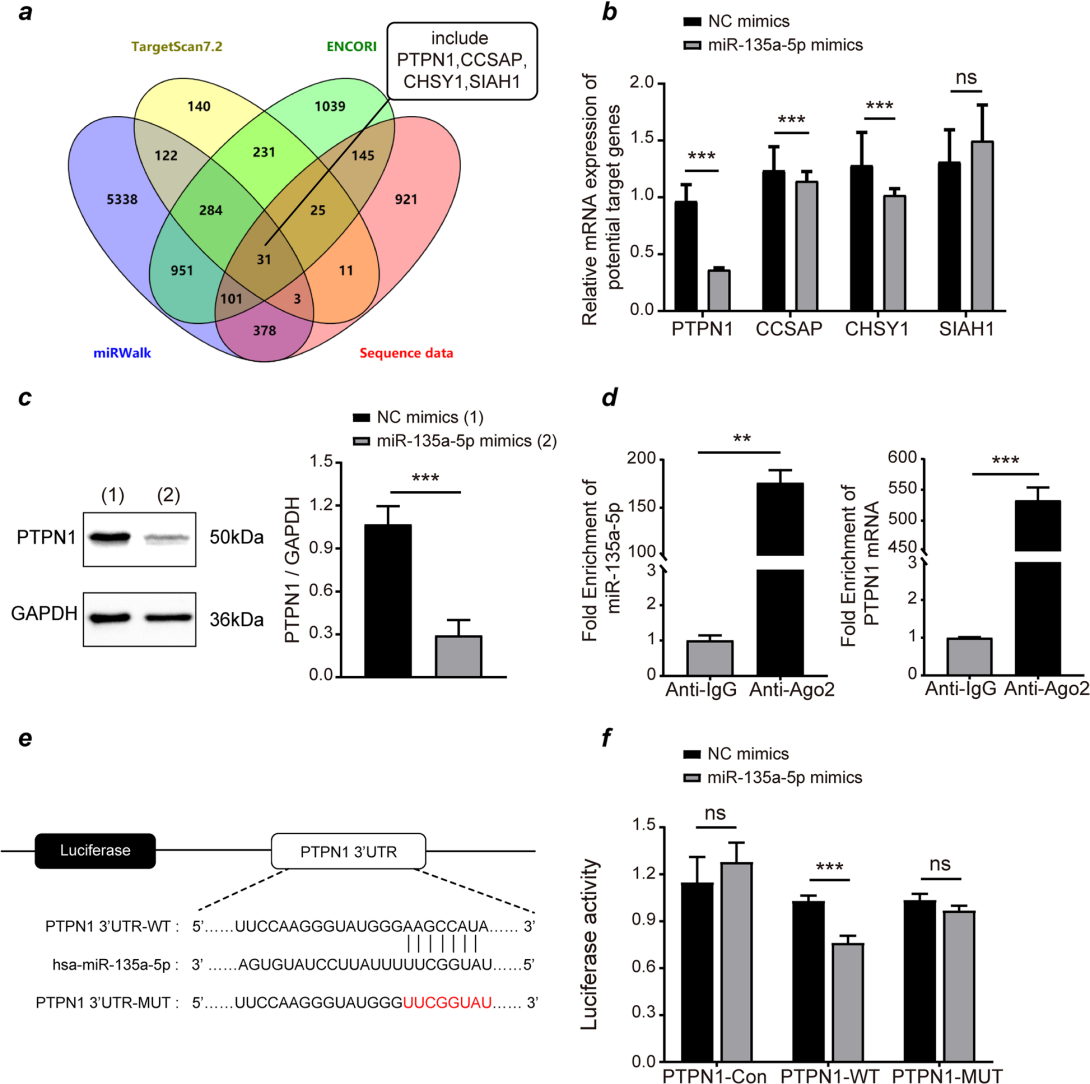
miR-135a-5p targets PTPN1 in HTR-8/SVneo cells**.** (**a**) Intersecting candidate miR-135a-5p target genes were identified using three independent miRNA target databases and downregulated differentially expressed genes in our sequencing dataset. (**b**) The expression of four putative target genes in HTR-8/SVneo cells were assessed following miR-135a-5p overexpression, revealing significant PTPN1 downregulation. (**c**) PTPN1 protein levels were reduced following miR-135a-5p overexpression in HTR-8/SVneo cells, with GAPDH as a normalization control. (**d**) Ago2 RIP-PCR revealed the preferential enrichment of miR-135a-5p and PTPN1 mRNA in samples precipitated with Ago2. (**e**) Alignment of miR-135a-5p with the predicted binding site in the PTPN1 3’-UTR. (f) HTR-8/Svneo cells were co-transfected with PTPN1 3’-UTR constructs and miR-135a-5p mimics or NC mimics, with the ratio of Firefly/Renilla luciferase activity then being quantified. Outcomes are means ± SD from three assessments. PTPN1: Protein Tyrosine Phosphatase Non-Receptor Type 1; Ago2-RIP: Ago2-RNA immunoprecipitation; UTR: untranslated region; ns: non-significant; NC: negative control. *P < 0.05, **P < 0.01, *** *P* < 0.001

Given that miRNAs bond onto 3′-UTR for target mRNAs to promote their degradation, we utilized TargetScan (V7.2), ENCORI, miRWalk, and downregulated differentially expressed genes from our previous sequencing data to predict miR-135a-5p target genes, revealing 31 overlapping genes (Fig. 5). Four of these intersecting genes, including PTPN1, CHSY1, SIAH1, and CCSAP, were correlated with tumor cell proliferation, migration, and invasion, and we therefore further confirmed their expression levels via RT-qPCR. We observed marked PTPN1 downregulation after miR-135a-5p overexpression within transcript levels and protein expression (*p* < 0.05, Fig. 5b-c). TargetScan database indicated that miR-135a-5p may inhibit PTPN1 expression through targeting the 3’UTR region of PTPN 1. Then we performed RIP experiments, which showed miR-135a-5p and PTPN1 to be enriched by Ago2 (*p* < 0.05, Fig. 5d), suggesting miR-135a-5p may bond onto 3’UTR for PTPN1. To more fully elucidate interactions between miR-135a-5p and PTPN1, a Dual-luciferase reporter assay was conducted revealing miR-135a-5p mimics transfection in HTR-8/SVneo cells decreased the luciferase function for PTPN1-wt but not PTPN1-mut reporter (p < 0.05, Fig. 5e-f). As such, PTPN1 represents a bona fide miR-135a-5p target gene in HTR-8/SVneo cells.

## Discussion

Here, we determined that miR-135a-5p can serve as an inhibitor of trophoblast proliferation, angiogenesis, invasion, and migration, suggesting that it can contribute to the incidence of unexplained SA and making it a promising biomarker associated with this negative pregnancy complication.

Accumulating evidence suggests that the abnormally expressed miRNAs are related to gestational diseases. For example, Ding et al. reported miR-27a-3p upregulation in villous tissue samples associated with recurrent miscarriages [17], while villous miR-98 upregulation has been observed in cases of missed abortion [36]. We similarly found miR-135a-5p to be upregulated in villous tissue samples associated with unexplained SA relative to those from NPs, suggesting a potential link between this miRNA and SA incidence. Chim et al. found that placental miRNAs were detectable within maternal plasma for extended periods of time, thus offering potential value as diagnostic biomarkers for various gestational diseases [37]. Consistently, multiple reports have demonstrated corresponding increases in the levels of placental and maternal plasma miR-155 and miR-210 in cases of preeclampsia. These data suggest the potential for these miRNAs as biomarkers for preeclampsia [38–41]. Shahidi et al. further found miR-146b and miR-520 h to be expressed at higher levels in plasma samples from RSA patients [42]. In line with our work, Hosseini et al. detected miR-135a upregulation in samples of maternal plasma associated with the early loss of pregnancy [29]. Given the ROC curve analyses of miR-135a-5p in villous tissues of unexplained SA, we speculate that miR-135a-5p can therefore offer value as a potential biomarker for this pregnancy complication. Future studies should thus be conducted comparing matched plasma and placental samples from individual unexplained SA patients in order to assess correlations in miR-135a-5p expression between these two compartments. Prospective cohort studies will also be necessary to confirm the utility of miR-135a-5p as a predictor or diagnostic biomarker in SA.

Trophoblasts are the primary cell type that composes the placental villous tissue, and they are necessary to support embryonic and fetal growth, shaping the overall function of the placenta [43–46]. As such, trophoblast impairment can compromise placental villous structure and functionality, potentially contributing to negative pregnant outcomes including preeclampsia, SA, and intrauterine growth restriction (IUGR) [47]. Here, we thus studied the impact of miR-135a-5p on growth and migratory activity in immortalized extravillous trophoblast cell line HTR-8/SVneo, which was originally isolated from healthy first-trimester villous explants. Our analyses suggested that overexpression of miR-135a-5p can inhibit proliferation, migration, invasion, and angiogenesis in HTR-8/SVneo cells. Moreover, overexpression of miR-135a-5p suppressed the expression of cyclin D1, cyclin B1, MMP2, MMP9, and vimentin protein within HTR-8/SVneo cells, whereas promote the expression of E-cadherin. Consequently, we propose a putative mechanism of miR-135a-5p in SA is causing extravillous dysfunction, impairing their anchoring of the uterine decidua and spiral artery remodeling. However, further studies are needed to be performed.

Current evidence suggests that inflammatory responses are involved in implantation and early pregnancy [48, 49]. In the context of ischemia-reperfusion injury, miR-135a-5p suppresses cardio-cerebral ischemic inflammatory damage [21, 22], and it has similarly been shown to hamper NLRP3 inflammasome activation and to attenuate associated inflammatory responses [31]. The upregulation of villous miR-135a-5p may contribute to SA by suppressing the inflammatory response during implantation.

The PTPN1 gene encodes PTP1B (Protein Tyrosine Phosphatase 1B) which was initially detected in human placental tissue but has since been shown to play a role in many essential cellular functions across different tissue types. PTP1B also plays roles in diseases such as cancer, cardiovascular disease, obesity, and diabetes [50–52]. In cancer, PTPN1 can act as an oncogene, suggesting that its inhibition may be therapeutically valuable [50]. Yu et al. posited that PTPN1 may act as an oncogene for promoting breast cancer cellular proliferation [53], with its overexpression can similarly enhance hepatocellular carcinoma cell malignancy and migratory activity [54], whereas PTP1B suppression promotes cell cycle arrest in pancreatic cancer cells in vitro [55]. How PTPN1 functions in trophoblasts remains incompletely understood, but our results suggest that it is a miR-135a-5p target gene within HTR-8/SVneo cells. As tumor cells and trophoblasts exhibit many similarities and we observed miR-135a-5p overexpression to inhibit PTPN1 expression within HTR-8/SVneo, we hypothesize that miR-135a-5p may impair proliferative, invasive, and migratory activity of trophoblasts via PTPN1.

## Conclusions

In summary, our data are consistent with a model wherein miR-135a-5p upregulation MAY play a role in miscarriage pathogenesis owing to its ability to influence the function of trophoblasts, thus offering promise as a biomarker for diagnosis unexplained SA. Mechanistically, our achievements suggest that the miR-135a-5p/PTPN1 axis can perform a key task in the incidence of SA. Future prospective cohort studies in larger sample size will be required to accurately validate the utility of miR-135a-5p as a prognostic or diagnostic biomarker associated with unexplained SA.

## Supplementary Information

**Additional file 1.**

**Additional file 2.**

## Data Availability

The Sequencing data sharing is not applicable to this article as no datasets were generated or analyzed during the current study. While other datasets used and/or analyzed during the current study are available from the corresponding author on reasonable request.

## Abbreviations

miRNA: microRNA
SA: Spontaneous abortion
NP: Normal pregnancy
USM: Unexplained sporadic miscarriage
URSA: Unexplained recurrent spontaneous abortion
PTPN1: Protein Tyrosine Phosphatase Non-Receptor Type 1
CCSAP: Centriole, Cilia and Spindle Associated Protein
CHSY1: Chondroitin Sulfate Synthase 1
SIAH1: Siah E3 Ubiquitin Protein Ligase 1
E-cad: E-cadherin
RT-qPCR: Real-time quantitative polymerase chain reaction
CCK-8: Cell counting Kit-8
EdU: 5-Ethynyl-2′-deoxyuridine
Ago2-RIP: Ago2-RNA immunoprecipitation
AUC: Area under the curve
BMI: Body mass index
NC: Negative control
UTR: Untranslated regions
