# Supplementary material for: MiR-135a-5p suppresses trophoblast proliferative, migratory, invasive, and angiogenic activity in the context of unexplained spontaneous abortion
Source: Reprod Biol Endocrinol. 2022 May 24;20:82. doi: 10.1186/s12958-022-00952-z (PMC9128262; doi:10.1186/s12958-022-00952-z)
Supplement: Supplementary file 2 — Additional file 2. [file 12958_2022_952_MOESM2_ESM.docx]

**Table S2. Clinical data of abortion patients**

|  | NP (n=50) | unexplained SA (n=50) | P. value |
| --- | --- | --- | --- |
| Maternal age, y | 31.7±5.13 | 31.6±4.98 | 0.943 |
| Gestational age, wk | 7.16±0.67 | 6.84±0.82 | 0.248 |
| BMI (Body mass index)  (Kg/M^2^) | 21.36±2.91 | 23.63±4.51 | 0.352 |
| History of gestation | 3.42±0.79 | 3.00±1.15 | 0.433 |

**Table S3. Transfection sequences of miR-135a-5p mimics, miR-135a-5p inhibitor, NC mimics and NC inhibitor**

| Items | Sequences (5’→3’) |
| --- | --- |
| miR-135a-5p mimics | UAUGGCUUUUUAUUCCUAUGUGA  ACAUAGGAAUAAAAAGCCAUAUU |
| NC mimics | Sense: UUCUCCGAACGUGUCACGUTT  Antisense: ACGUGACACGUUCGGAGAATT |

**Table S4. Primers sequences for RT-qPCR and Argonaute 2-RNA immunoprecipitation**

| MiRNA/Gene | Primers sequences |
| --- | --- |
| miR-135a-5p^*^ | Forward: AAGCGACCTATGGCTTTTTATTCCT |
| U6 | Forward: TGGAACGCTTCACGAATTTGCG  Reverse: GGAACGATACAGAGAAGATTAGC |
| PTPN1 | Forward: CAGAAAAGCAGGGGTGTCGT  Reverse: CCAAAGTCAGGCCATGTGGT |
| PTPN1-RIP | Forward: TCCTGGTACAGCAGGGTCTT  Reverse: TGTTGACTTGGAATGGCGGA |
| CHSY1 | Forward: TTGTCTGGGCGTTTCGACAT  Reverse: CTGCATGTCGGCTTTAGGGT |
| SIAH1 | Forward: CACCCATCTGTCTGCCAACC  Reverse: AGTGTTACTACATGCAACTGTTTCC |
| CCSAP | Forward: GAGACTGACAAATCACCCACCA  Reverse: TCTGTTCTTGGCTCGTAATGCT |
| β-actin | Forward: TGGCACCCAGCACAATGAA  Reverse: CTAAGTCATAGTCCGCCTAGAAGCA |

RT-qPCR: Real-time quantitative polymerase chain reaction; miR-135a-5p: microRNA-135a-5p; PTPN1: Protein Tyrosine Phosphatase Non-Receptor Type 1; CHSY1: Chondroitin Sulfate Synthase 1; SIAH1: Siah E3 Ubiquitin Protein Ligase 1; CCSAP: Centriole, Cilia and Spindle Associated Protein.

* miR-135a-5p downstream primers are from MRQ primer in mir-x ™ miRNA first strand synthesis of Takara reverse transcription kit. Their sequences are unpublished.
